# A Novel BODIPY Quaternary Ammonium Salt-Based Fluorescent Probe: Synthesis, Physical Properties, and Live-Cell Imaging

**DOI:** 10.3389/fchem.2021.650006

**Published:** 2021-03-12

**Authors:** Peng Deng, Fuyan Xiao, Zhou Wang, Guofan Jin

**Affiliations:** ^1^ The People’s Hospital of Danyang, Affiliated Danyang Hospital of Nantong University, Zhenjiang, China; ^2^ School of Pharmacy, Jiangsu University, Zhenjiang, China; ^3^ College of Vanadium and Titanium, Panzhihua University, Panzhihua, China

**Keywords:** BODIPY, quaternary ammonium salt, HeLa cells, *in vitro* imaging, *in vivo* imaging

## Abstract

The development of biological fluorescent probes is of great significance to the field of cancer bio-imaging. However, most current probes within the bulky hydrophobic group have limited application in aqueous medium and restricted imaging under physiological conditions. Herein, we proposed two efficient molecules to study their physical properties and imaging work, and the absorption and fluorescence intensity were collected with varying ions attending in aqueous medium. We enhance the water solubility through the quaternization reaction and form a balance between hydrophilic and hydrophobicity with dipyrrome-theneboron difluoride (BODIPY) fluorophore. We introduced pyridine and dimethylaminopyridine (DMAP) by quaternization and connected the BODIPY fluorophore by ethylenediamine. The final synthesized probes have achieved ideal affinity with HeLa cells (human cervical carcinoma cell line) in live-cell imaging which could be observed by Confocal Microscope. The probes also have a good affinity with subcutaneous tumor cells in mice in *in vivo* imaging, which may make them candidates as oncology imaging probes.

## Introduction

Small molecular probes with a high fluorescence signal are of use in cancer imaging development and play important roles in the study of biological activity and metabolism in cancer disease treatment (Gonzalez-Vera et al., 2020; Zhao et al., 2020; Zheng et al., 2020). There are many members in traditional fluorescent probes family, such as fluorescein, rhodamine, and some other potential probes (Chen et al., 2020; Ren et al., 2020; Zhong et al., 2020). With the continuous emergence of probes, small molecular fluorescent probes cut a striking figure in this field. One of these probes, named BODIPY, has become the key subject in the view of researchers, due to its excellent photophysical properties and the advantage of easy modification (Liu et al., 2020; Wang H. et al., 2020). However, there are still many problems to be solved for the structural modification of BODIPY, such as its highly structural complexity and poor water solubility. Based on this, a simple and convenient preparation method from easily available raw materials also needs to be proposed.

Aqueous systems are essential in life processes and the global environment. But traditional BODIPY dyes are only soluble in organic solvents, and their low solubility mean they have limited application in biological fields. To this end, H.J. Worries introduced a chlorosulfonic acid group to the core structure in 1985, which markeda key first step to advancing water solubility (Choi et al. 2018). Inspired by this, some related work on improving solubility has been carried out. The water solubility of BOPIDY can be improved by introducing different types of hydrophilic groups such as sulfonate (Koh et al., 2019), phosphonate, and quaternary ammonium salt (Mao et al., 2020; Zhou et al., 2020). Common water-soluble modification occurs in the core structure or boron atom center, and the 2, 6 or 3, 5 sites are common modification sites. Even though many works are studying the exploration of hydrophilicity, it remains superficial, which has caused certain obstacles to the imaging application of compounds. Research on the development of fluorescent probes with a simplified structure and enhanced water solubility is still in its infancy stage and more work needs to be done in the future.

As a noticeable probe synthesized since 1968, BODIPY has many new biological applications which have been gradually discovered and gained more and more attention (Wang R. et al., 2020; Xu et al., 2020). Live cell imaging is a great example of these applications (Guasch-Ferre et al., 2020; Wang F. et al., 2020). It is an important practice in biomedical study for the analysis of the functional and pathological of cells and tissues and clinical diagnosis. Water soluble modification opens up a new avenue for bioactivity research, including cell imaging and research on water-soluble probes for imaging, the study of which has gradually increased (Callmann et al., 2020; Liang et al., 2020). For example, Jin et al. synthesized a new water-soluble compound (4,4-di-(4′-methylmercaptophenoxy)-8-(N-methylpyridinium-2-yl)-1,3,5,7-tetramethyl-4-bora-3a,4a-diaza-s-indacene) and successfully applied it to imaging in living cells (Wegner and Zimmermann, 2004; Khailova et al., 2020; Tang et al. 2020).

In this paper, we report two novel water-soluble fluorescent probes, BDP-1 and BDP-2, for live cell imaging. The unique BODIPY core with strong fluorescence properties provides new possibilities for cell imaging and metabolism research. In the probe design work, the balance between hydrophilicity and lipophilicity is comprehensively considered. The hydrophobicity fluorescein pyrrole nucleus acts as the fluorophore. The side chain ethylenediamine bridge group is introduced to adjust its water-lipid balance. Finally, the N-choloropyridinium with a positive charge moiety group acts as a water-soluble improving part. In this paper, the concise synthetic strategy of novel BODIPY probes has been proposed and the optical properties in the presence of different ions are also explored. The probes have achieved ideal non-specific affinity with HeLa cells (human cervical carcinoma cell line) in live cell imaging and a good non-specific affinity with subcutaneous tumor cells in mice in *in vivo* imaging.

## Materials and Methods

### General Materials

The acetonitrile solvent used in the reaction was distilled by calcium hydride in advance. In the characterization part, the ^1^H and ^13^C spectra were recorded on Bruker Avance spectrometer (400 MHz for ^1^H, 101 MHz for ^13^C) in CD_3_OD with the Me_4_Si at chemical shifts δ 0.00 ppm as standard to characterize the structures. Ultraviolet-visible (UV-vis) and fluorescence spectra were recorded on a UV-2550 spectrophotometer and Shimadzu RF-5301PCS spectrofluorophotometer, respectively, at room temperature.

### Synthesis

Synthesis of 7-chloro-2-ethyl-5,5-difluoro-1,3-dimethyl-10-phenyl-5H-4l4,5l4-dipyrrolo[1,2-c:2′,1′-f] [1,3,2]diazaborinine (1).

The compound 1 was synthesized from 2-chloro-5-benzoyl-pyrrole (0.8 g, 4.0 mmol), POCl_3_ (2 ml), and 2,4-dimethyl-3-ethylpyrrole (1.8 g, 15.0 mmol) in dichloromethane through stirring for 24 h at room temperature. Neutralization by NaHCO_3_ was carried out to obtain the intermediate. Et_3_N (2 ml) was added into the intermediate in toluene and BF_3_·OEt (2 ml) was added by stirring for 7 h at 100°C. Neutralization by NaHCO_3_ was carried out again and purification by column chromatography to gain 1 was used (0.5 g, 41%).

Synthesis of (Z)-N1-(1-(difluoroboranyl)-5-((4-ethyl-3,5-dimethyl-2H-pyrrol-2-ylidene) (phenyl)methyl)-1H-pyrrol-2-yl)ethane-1,2-diamine (2).

(Z)-2-chloro-1-(difluoroboranyl)-5-((4-ethyl-3,5-dimethyl-2H-pyrrol-2-ylidene) (phenyl)methyl)-1H-pyrrole (0.3 g, 0.8 mmol) was mixed with the dry acetonitrile solution of ethane-1,2-diamine (0.1 g, 1.7 mmol) with the attendance of triethylamine (0.2 g, 1.3 mmol) and stirred for 6 h at room temperature. The reaction mixture was evaporated and purified by silica gel column chromatography to obtain 2 in red powder (0.3 g, 91%). ^1^H NMR (400 MHz, CDCl_3_) δ 7.42−7.40 (m, 3H), 7.31−7.29 (m, 2H), 6.46 (d, *J* = 4.4 Hz, 1H), 5.92 (d, *J* = 4.8 Hz, 1H), 3.409 (s, 2H), 3.00 (t, *J* = 6.0 Hz, 2H), 2.47 (s, 3H), 2.35−2.29 (dd, *J* = 7.6, 14.8 Hz, 2H), 1.37 (s, 3H), 0.99 (t, *J* = 7.6 Hz, 3H). ^13^C NMR (100 MHz, CDCl_3_) δ 159.8, 144.9, 135.2, 133.3, 133.1, 132.4, 132.3, 130.0, 129.6, 128.3, 128.0, 106.0, 46.8, 41.4, 29.7, 17.1, 15.0, 11.9, 11.6. ITMS (ESI) calculated for C_21_H_25_BF_2_N_4_ [M + H]^+^ m/z 383.2218; found 383.2345.

General synthesis procedure of 1-(2-((2-((8-ethyl-5,5-difluoro-7,9-dimethyl-10-phenyl-5H-5l4,6l4-dipyrrolo[1,2-c:2′,1′-f] [1,3,2]diazaborinin-3-yl)amino)ethyl)amino)-2-oxoethyl)pyridin-1-ium chloride (BOD-1) and 4-(dimethylamino)-1-(2-((2-((8-ethyl-5,5-difluoro-7,9-dimethyl-10-phenyl-5H-5l4,6l4-dipyrrolo[1,2-c:2′,1′-f][1,3,2]diazaborinin-3-yl)amino)ethyl)amino)-2-oxoethyl) pyridin-1-ium chloride (BOD-2).

Oxalyl chloride (0.2 ml, 3.0 mmol) was slowly dropwised to the previously synthesized 2 (0.3 g, 1.0 mmol) under ice bath temperature with 5 ml acetonitrile as a solvent. The reaction was over after 10 min and purified by silica gel column chromatography to obtain 3. The pyridine (3 ml, 37 mmol) or DMAP (0.08 g, 0.7 mmol) was mixed with 3 (0.05 g, 0.1 mmol) in a pressure tube and the resulted mixture was heated at 50°C for 8 h. From that, DMAP was reacted in 2 ml acetonitrile, while pyridine acted as a solvent. Then the reaction was evaporated by rotary evaporation and washed with 3 ml EtOAc and Petroleum ether (1: 3) to obtain pure crimson solid.

#### Synthesis Procedure of BOD-1

The pyridine (3 ml, 37 mmol) was mixed with 3 (0.05 g, 0.1 mmol) according to the general procedure to obtain pure powder BDP-1 (0.04 g, 68.2%). ^1^H NMR (400 MHz, CD_3_OD) δ 8.62 (d, *J* = 4.4 Hz, 2H), 8.10 (t, *J* = 6.8 Hz, 1H), 7.49 (t, *J* = 2 Hz, 3H), 7.31−7.29 (m, 2H), 6.50 (d, *J* = 4.8 Hz, 1H), 6.20 (d, *J* = 4.8 Hz, 1H), 5.47 (s, 2H), 3.56 (s, 4H), 3.34 (m, 2H), 3.07 (d, *J* = 10 Hz, 1H), 2.44 (s, 3H), 2.37 (d, J = 7.6 Hz, 2H), 1.39 (s, 3H), 1.03 (t, *J* = 7.6 Hz, 3H). ^13^C NMR (100 MHz, CD_3_OD) δ 165.1, 160.3, 148.3, 146.1, 146.0, 143.5, 135.2, 133.1, 132.8, 132.4, 131.1, 129.5, 128.8, 127.9, 127.5, 121.4, 106.8, 61.6, 43.0, 39.3, 16.6, 14.1, 10.7, 10.4. HR-MS (FAB) calculated for C_28_H_31_BClF_2_N_5_O [(M-BF2)^+^-Cl^−^] m/z, 454.26014, observed 454.25972.

#### Synthesis Procedure of BDP-2

This was carried out according to the general procedure to obtain pure crimson powder BDP-2 (0.03 g, 47.4%). ^1^H NMR (400 MHz, CDCl_3_) δ 8.13 (d, *J* = 6.2 Hz, 2H), 8.01 (s, 1H), 7.48 (d, *J* = 4.7 Hz, 3H), 7.41 (d, *J* = 3.5 Hz, 2H), 7.29 (d, *J* =4.3 Hz, 2H), 6.99 (d, *J* = 6.5 Hz, 2H), 6.88 (d, *J* = 5.6 Hz, 2H), 6.53−6.44 (m, 1H), 6.23 (s, 1H), 3.54 (d, *J* = 14.2 Hz, 4H), 3.25 (s, 6H), 2.43 (d, *J* = 5.6 Hz, 3H), 2.38 (dd, *J* = 7.6 Hz, 2H), 1.38 (d, *J* = 4.8 Hz, 3H), 1.00 (t, *J* = 7.6 Hz, 3H). ^13^C NMR (100 MHz, CD_3_OD) δ 166.9, 157.7, 156.6, 147.8, 142.9, 138.7, 135.2, 134.3, 133.1, 132.5, 130.8, 129.5, 128.3, 127.9, 122.3, 107.1, 106.8, 58.1, 43.0, 38.9, 38.8, 16.6, 14.1, 10.7, 10.4. HR-MS (FAB) calculated for C_30_H_36_BClF_2_N_6_O [(M-BF2)^+^-Cl^−^] m/z, 497.30234, observed 497.30167.

### Photophysical Properties and Sensing of Target Ions

Ultraviolet-visible (UV-vis) and fluorescence spectra were recorded on a UV-2550 spectrophotometer and Shimadzu RF-5301PCS spectrofluorophotometer, respectively, at room temperature. The mother liquor for sensing in aqueous solution was prepared at concentration of 5 mM and diluted into the desired concentration. The spectra data was collected by the above instruments under different preset concentrations with the absence and attendance of eight target ions after mixing evenly.

### Transmission Electron Microscope Analysis

Transmission electron microscopy of compounds (10 μM) BDP-1 and BDP-2 was conducted in methanol. The sample of the configured electron microscope was dissolved in methanol solution, and the pictures were collected by transmission electron microscope (Japan Electronics, JEM-1400plus) at room temperature.

### Cytotoxicity Analysis

HCT-116, Hela, and normal liver L-02 cells were screened for *in vitro* cytotoxicity, and all were purchased from the American type culture collection (United States). HCT-116 and Hela cells were routinely cultured in RPMI-1640, while L-02 cells were routinely cultured in DMEM. 10% fetal bovine serum (FBS, purchased from Hangzhou Sijiqing Biological Engineering Materials Co., Ltd.) was added to the medium, and the cells were sub-melted in a humidified atmosphere at 37°C and 5% CO_2_. These cells were monitored daily and maintained at 80% cell density.

MTT cancer cells (HCT-116 and Hela) and normal human lung L-02 were tested for the cytotoxicity of each cell line in the logarithmic growth phase. All cells were seeded on 96-well plates at a rate of 106 cells per well. Then they were treated with berberine or compounds (BDP-1, BDP-2) at different concentrations, and the samples were tested for 24 h. Supernatant was dissolved in 100 ml DMSO and shaken for 10 min. The optical density of the sample was measured at 490 nm with a microplate photometer. Cell viability was expressed as the percentage change in absorbance relative to the control value.

### Hela Cervical Cancer Live Cell Imaging

Hela Cells were incubated with different BODIPY derivatives, BDP-1 and BDP-2 (5 mM, 2 μL), for 1 h at 37°C after good cultivation and washing. The culture medium was separated and discarded and treated with PBS afterward; the stained cells were then observed under the confocal laser scanning microscope with the emission wavelengths between 500–510 nm. Hela cells were also stained with DAPI (5 μg/ml) under the same operating procedures as the control group and images were collected.

### *In vivo* Experiment

Animal studies were conducted under institutional approval (Laboratory Animal Center of Jiangsu University, Zhenjiang, China). Two mouse components weighing 21 g and 20 g were used for *in vivo* imaging experiments. BDP-1 (20 μM) and BDP-2 (20 μM) were added by intraperitoneal injection under fasting conditions and fluorescence was collected after 30 min of exposure.

## Result and Discussion

### Chemistry

**SCHEME 1:**
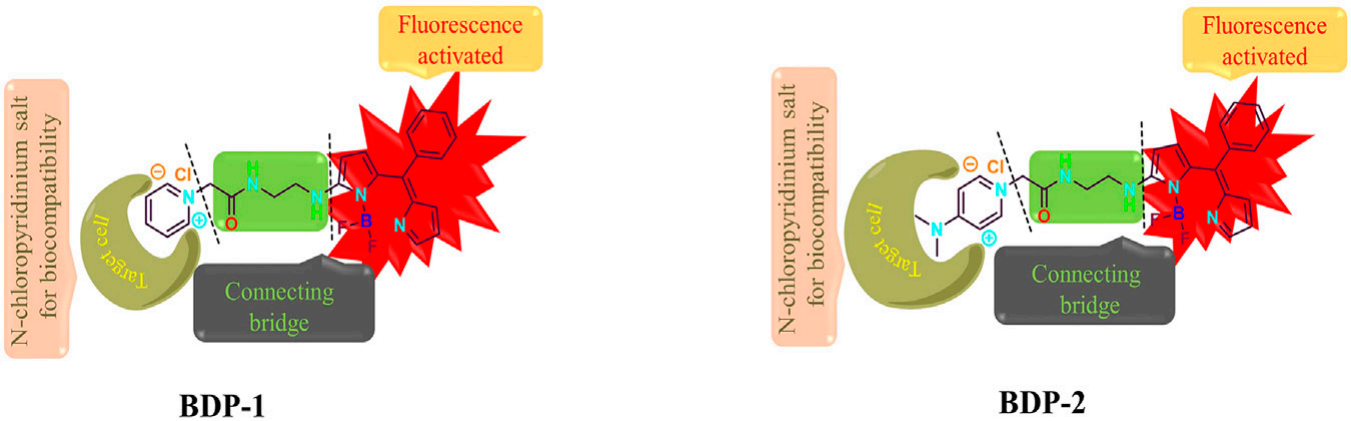
Chemical structure design and mechanism.

For the design, the fluorescent excimer is the (Z)-1-(difluoroboranyl)-2-((4-ethyl-3,5-dimethyl-2H-pyrrol-2-ylidene) (phenyl)methyl)-1H-pyrrole, and the BF_2_ unit inside this group can generate the intense emission band from S_1_ to S_0_ transition through the flow of electrons inside the molecule. As shown in Scheme 1, the green carbonyl amine part in the middle acts as a water-solubility part which is a vital bridge between the fluorescence core and the hydrophilic functional part. The hydrophilic pyridine moiety plays an important role in improving the biocompatibility and water solubility of the compound and it produces a marked effect by forming a super-hydrophilic quaternization structure. In addition, the positive charge enriches the target anion response in aqueous medium. Meanwhile, the oxygen-enriched and nitrogen-rich part in the structure as acts as a hydrogen bond donor or acceptor, which is beneficial to the improvement of live cell imaging. In summary, the powerful combination of the three parts provides unprecedented new ideas for the structural modification of BODIPY, and also adds color to the application of fluorescent probes for cancer cell imaging.

**SCHEME 2:**
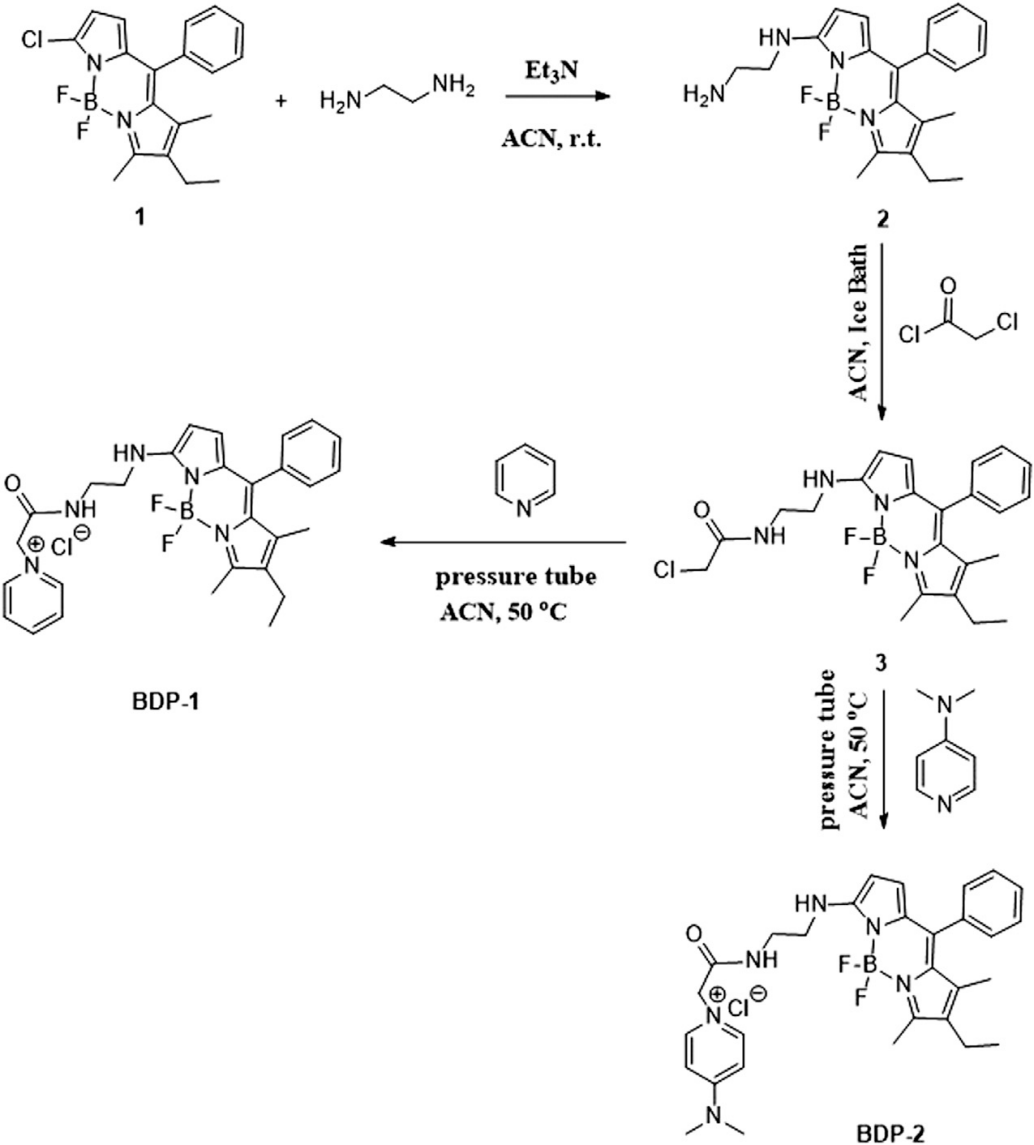
Synthetic route to the water-soluble N-choloropyridinium BODIPYs.

As shown in Scheme 2, the probes were synthesized in a few short steps. The compound 1 was synthesized from 2-chloro-5-benzoyl-pyrrole and 2,4-dimethyl-3-ethylpyrrole with the attendance of POCl_3_ in dichloromethane, with triethylamine and boron trifluoride etherate subsequently added (Suzuki et al., 2006; Carrascal et al., 2019; Li et al., 2019). We synthesized BDP-1 and BDP-2 starting from compound 1 followed by nucleophilic substitution with ethylenediamine and subsequently by acyl chlorination with chloroacetyl chloride to obtain intermediate 3. The intermediate 3 was quaternized with pyridine and 4-dimethylaminopyridine to improve the biocompatibility and increase the water solubility of the products.

### Photophysical Properties

**FIGURE 1:**
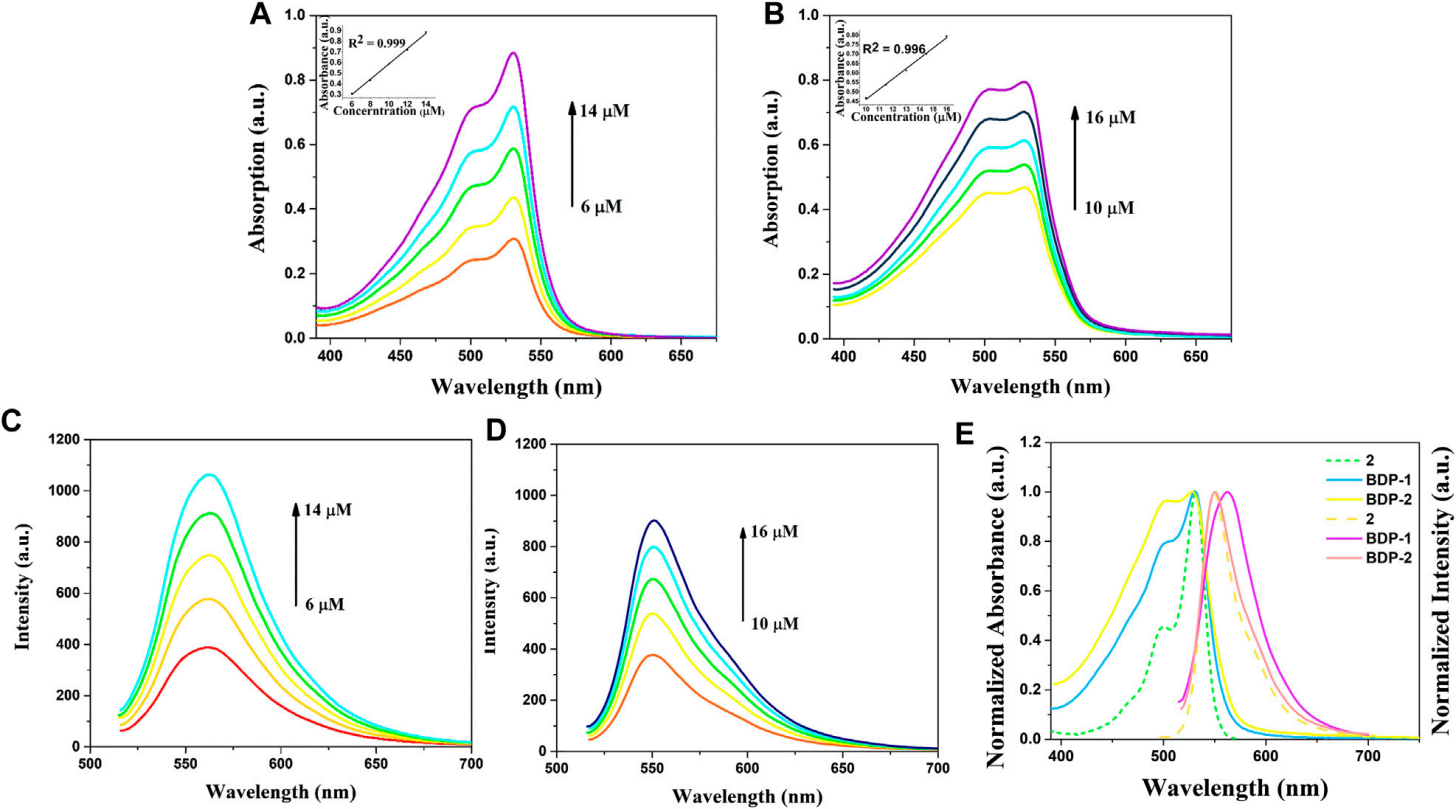
Absorption and emission spectra of BDP-1 and BDP-2. Absorption spectra of **(A)** BDP-1 and **(B)** BDP-2 in methanol at 25°C. Emission spectra of **(C)** BDP-1 and **(D)** BDP-2 in methanol at 25°C. (concentration BDP-1: 6, 8, 10, 12, 14 μM; BDP-2: 10, 11.5, 13, 14.5, 16 μM). **(E)** Normalized spectrum of 2, BDP-1 and BDP-2. Insert: The absorption changes upon the increasing concentration.

The optical spectra were also characterized in methanol at room temperature for its photophysical properties in Figure 1 and Table 1. The absorption spectrum data of BDP-1 and BDP-2 were collected in the concentration range from 6 to 14 μM and 10 to 16 μM, respectively, to keep it at an applicable range according to Lambert Beer's law. As shown in Figure 1, the absorption band of BDP-1 was similar in shape and peaks to BDP-2, retaining the maximum absorptions at 530 and 528 nm, respectively. The fluorescence spectra of BDP-1 showed maximum at 560 nm, although it showed a slightly greater red shift than that of BDP-2 and 2 (λ_em_ = 550 nm). This phenomenon may indicate that the fluorophore is served by the BODIPY core, and the introduced pyridine group does not negatively affect the fluorescence effect of the compound while providing an indicator of its hydrophilicity. Therefore, intensive fluorescence and proper hydrophilic-lipophilic equilibrium can serve as candidates for excellent cell imaging.

**TABLE 1:** Absorption and emission data.^a^

Compound	λ_max,abs_ (nm) (ε/10^5^ L·mol^−1^·cm^−1^)	λ_max,emi_ (nm)
2	530 (0.300)	550
BDP-1	530 (0.717)	560
BDP-2	528 (0.544)	550

^a^ Measurements were performed in MeOH unless otherwise noted. The excitation wavelength is 500 nm for 2, BDP-1, and BDP-2.

**TABLE 2:** Cytotoxicity analysis of BDP-1 and BDP-2 with the drug berberine as reference.

Comp.	IC_50_ (μM) ± SD		
	HCT-116	Hela	L-02
BDP-1	125.62 ± 3.70	110.46 ± 8.43	28.69 ± 8.73
BDP-2	103.83 ± 2.98	97.28 ± 11.93	33.78 ± 5.16
Berberine	29.47 ± 9.19	21.26 ± 3.11	>150

**FIGURE 2:**
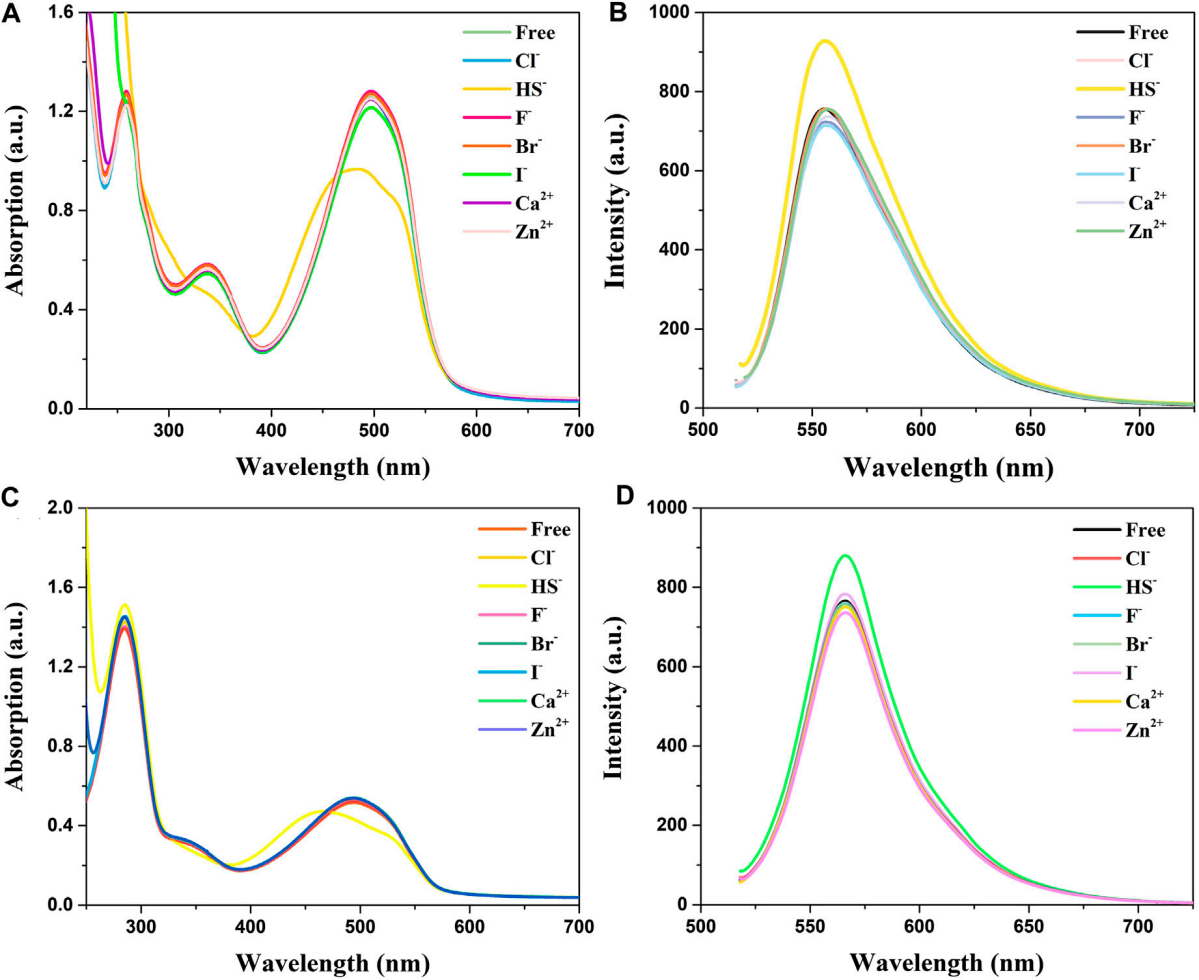
Absorption and fluorescence responses of probes to ions (100 μM) in aqueous solution. **(A)** Absorption and **(B)** Fluorescence spectra of BDP-1 (10 μM); **(C)** Absorption and **(D)** Fluorescence spectra of BDP-2 (10 μM). (1) Free; (2) Cl^−^; (3) NaHS; (4) F^−^; (5) Br^−^; (6) I^−^; (7) Ca^2+^; (8) Zn^2+^. Data were recorded 10 min after the addition of different ions.

Absorption and emission studies for ion response experiments were carried out and the result was shown in Figure 2. The results show that the compounds spectra did not change after the addition of of many ions (Cl^−^, F^−^, Br^−^, I^−^, Ca^2+^, and Zn^2+^), while the absorption peaks of HS^−^ ions are conspicuously blue-shifted from 500 to 450 nm accompanied by a significant decline in absorption spectra. Analysis of absorption spectroscopy results showed that BDP-1 has a more obvious effect on HS^−^ than other ions and produces a more obvious decrease in absorbance than BDP-2. The fluorescence emission spectrum recording situation is similar to the absorption spectrum which responds to H_2_S distinctly. Surprisingly, in the presence H_2_S, the fluorescence appears to increase slightly and differ from the addition of other tested ions. In the end, we deduce the reason for the fluorescence enhancement may be that the ionization of the active ions in water hinders the electron flow between the pyridine group and the fluorophore in the structure, and then affects the intramolecular electron transfer. And in order to prove this hypothesis, the following experiment was conducted.

**FIGURE 3:**
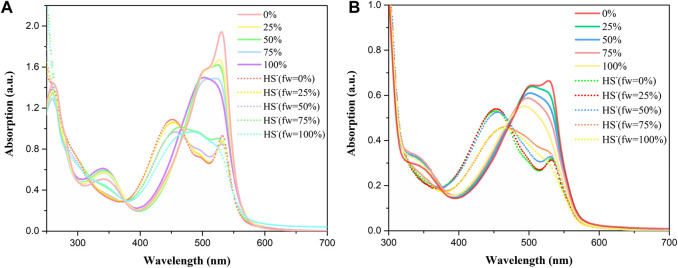
Absorption spectra at different fw (water/methanol) of **(A)** BDP-1 (10 μM) and **(B)** BDP-2 (10 μM) at 25°C (solid line), with the addition of NaHS at 25°C (short dot line).

In addition, the optical response of BDP-1 and BDP-2 to HS^−^ obviously depended on the volume fraction of water (fw) in the water/MeOH mixture which responded under all circumstances. As shown in Figure 3 (solid line), the methanol-dissolved BDP-1 maintained a sharper peak at 528 nm than that of the full water solvent with a blunt peak at 500 nm. It could be seen that, as the fw value gradually grows from 50 to 100%, the property of probes changed a lot in peak shape and maximum absorbance. On the other hand, after adding HS^−^ (short dot line), the spectrum changes obviously in morphology and peaks. The methanol dissolved BDP-1 still maintained a small peak at 528 nm with an evident blueshift from 528 nm to 450 nm with an increase of fw value. In detail, for the HS^−^-activated absorption transition, both the morphology and the distribution of absorption peaks showed a gradual reduction to lower and this phenomenon can be clearly reflected in pure water (fw = 100%). Furthermore, BDP-2 has a similar situation, with the strongest response changes in pure water. Therefore, in the presence of different volume fractions of water (fw), it has a stable response activity to H_2_S as expected, so it has great potential for application research. This phenomenon could also prove our previous hypothesis that ionization in aqueous solution induces fluorescence enhancement.

In addition, to further observe the morphology of the quaternized compounds, the structure characterization was also carried out by transmission electron microscope (TEM) characterization in methanol at 25°C, and the self-assembly BDP-1 and BDP-2 structures were clearly presented. As can be seen in the image (Supplementary Figure S10), the compound is in a state of aggregation in the solution. This aggregation may be inferred to be caused by the positive charge of the compound itself resulting in it layering itself on top of each other; thus, the aggregation becomes like the picture.

**FIGURE 4:**
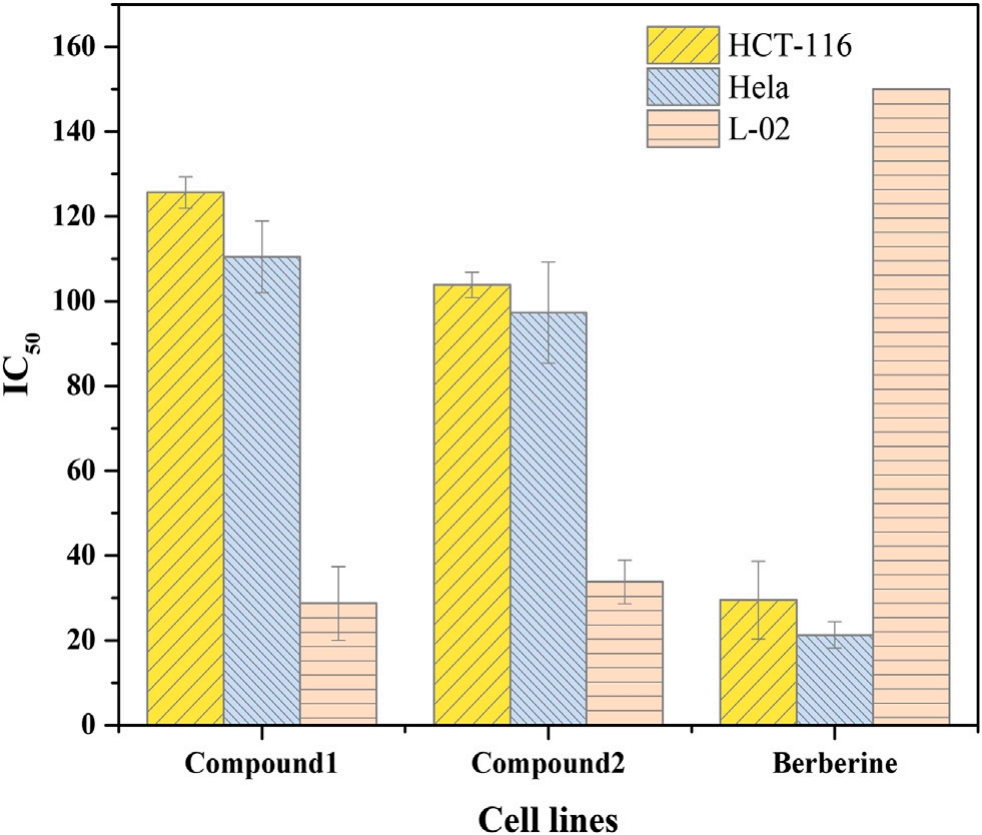
Cytotoxicity analysis of BDP-1 and BDP-2 with the drug berberine as reference.

It is interesting to analyze anti-proliferation as shown in Figure 4. IC_50_ values of 125.62 ± 3.70 and 103.83 ± 2.98 μM were observed for BDP-1 and BDP-2 in HCT-116 cells at 37°C, respectively IC_50_ values of 110.46 ± 8.43for 5 and 97.28 ± 11.93 μM for 6 in HeLa cell line were observed. For a non-cancerous lung cell line (L-02), the toxicity of 5 (28.69 ± 8.73 μM) and 6 (33.78 ± 5.16 μM) is not ideal compared to the reference drug and should be improved in further study. Therefore, reducing the toxicity to normal cells and adjusting the balance between the water-solubility and bioactivity is also a required direction.

The BDP-1 and BDP-2 presents obvious aggregation and displayed good stability in methanol solvent under the 200 nm scale. Meanwhile, the presence of positive charges in the structure leads to the stacking of layers. It could also infer that it keeps the balance between the hydrophilicity and lipophilicity of the compounds to achieve amphiphilicity, which may lead to this polymerization.

**FIGURE 5:**
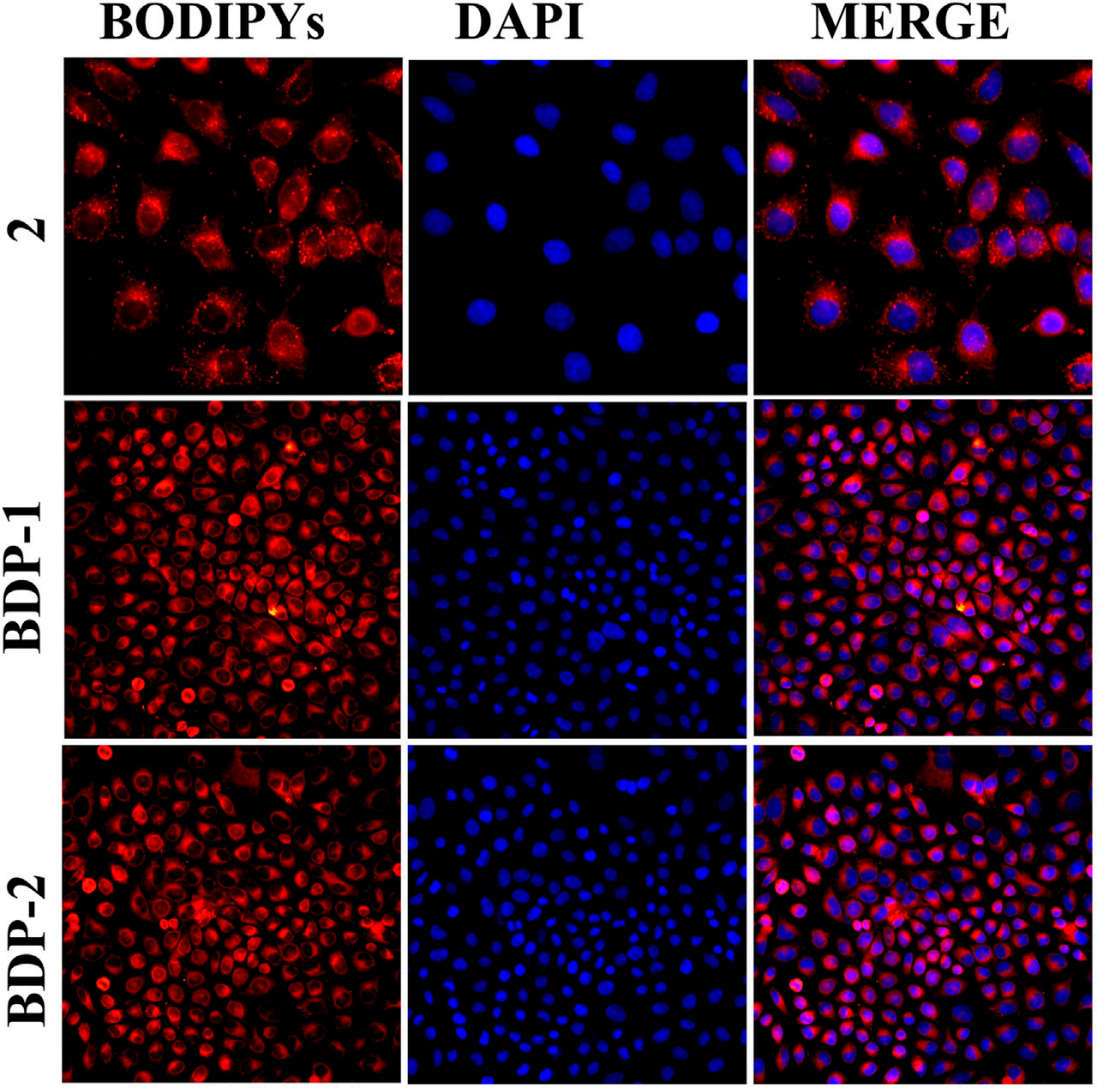
Cell imaging stained with samples under different compounds: compound 2, BDP-1, and BDP-2 treatment.

Moreover, the fluorescence performance in cell imaging means a lot for the application of probes in the fluorescent family. HeLa cells were incubated with BODIPYs for 30 min for cell imaging study. Then the imaging results were shown in Figure 5, and it could be directly observed that both BDP-1 and BDP-2 were uptaken by the HeLa cell membrane with desired fluorescence imaging. Both BODIPY derivatives show higher fluorescence than the non-quaternized compound 2. It could be deduced that the compounds were absorbed into the cell membrane and then firmly targeted to the cells due to the positive charge and the counterpoise of hydrophilic and lipophilic. Then they emit strong fluorescence in intracellular structures.

**FIGURE 6:**
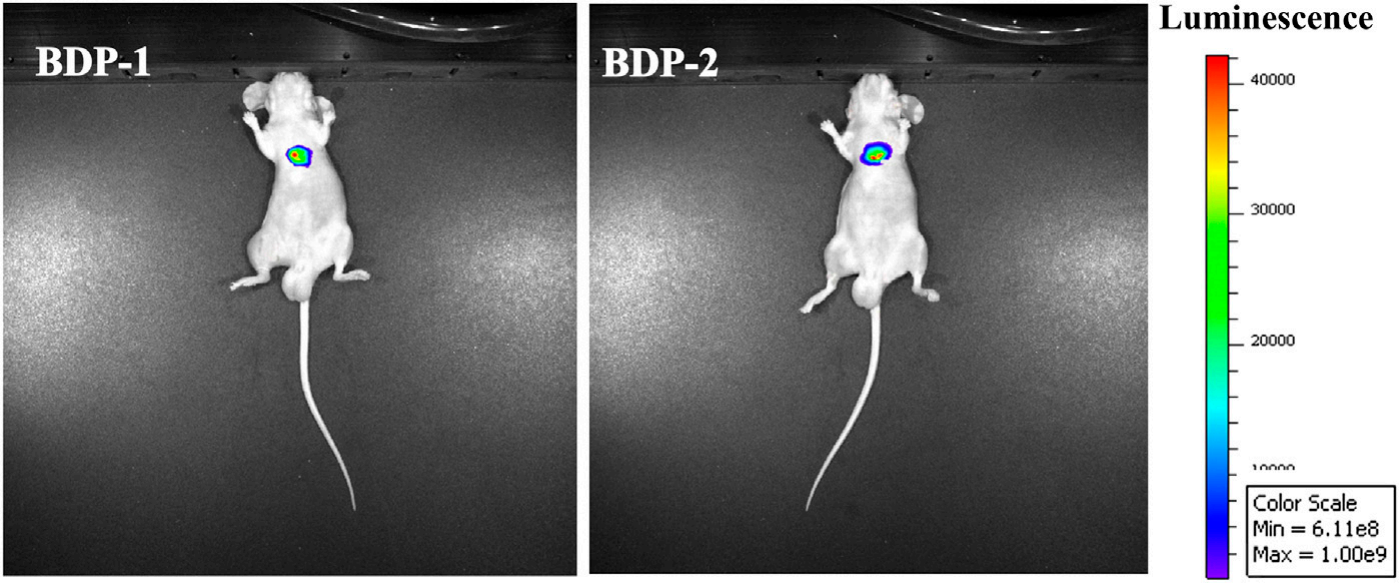
*In vivo* fluorescence images of mice after injection of BDP-1 (10 mM) and BDP-2 (10 mM).

*In vivo* fluorescence experiments in mice were designed and executed. BDP 1 and BDP 2 (10 mM) were intraperitoneally injected under fasting conditions in mice. After 40 min of exposure, the fluorescence intensity of the tumor tissue was detected. As shown in Figure 6, *in vivo* fluorescence is generated in subcutaneous tumors. It is reasonable that the fluorescence intensity of the two compounds is high and the fluorescence brightness is sensitive. Finally, it can be observed that the precise generation and penetration of fluorescence in subcutaneous tumors have the potential for further medical imaging applications.

## Conclusion

In conclusion, we designed and prepared two water-soluble small molecule probes that have ideal fluorescence intensity and good potential for cancer cell imaging applications. Traditional small-molecule fluorophores have the shortcomings of low water solubility and weak fluorescence, however, the BODIPYs synthesized in this paper could not only generate high intensity fluorescent, but also solve the problem of water solubility. What’s more, they could also serve as potential probes in aqueous solution. The application of cell imaging contributes to intracellular visualization for biological study. The newly synthesized probes with excellent photophysical properties have a broad development prospect, which lay a foundation for further research on water-soluble ion probes.

## Data Availability

The original contributions presented in the study are included in the article/Supplementary Material, further inquiries can be directed to the corresponding author.
