# Supplementary material for: A Novel BODIPY Quaternary Ammonium Salt-Based Fluorescent Probe: Synthesis, Physical Properties, and Live-Cell Imaging
Source: Front Chem. 2021 Mar 12;9:650006. doi: 10.3389/fchem.2021.650006 (PMC7994363; doi:10.3389/fchem.2021.650006)
Supplement: Supplementary file 1 [file datasheet1.docx]

**Supporting Information**

**Table of Contents**

**A novel BODIPY quaternary ammonium salt-based fluorescent probes: Synthesis, physical property and live-cell imaging**

Peng Deng^1^, Fuyan Xiao^3^, Zhou Wang^2^, and Guofan Jin ^3,*^

^1^The People’s Hospital of Danyang, Affiliated Danyang Hospital of Nantong University, Zhenjiang, 212300, P.R. China

^2^College of Vanadium and Titanium, Panzhihua University, 617000, P.R. China

^3^School of Pharmacy, Jiangsu University, Zhenjiang 212013, P.R. China
*****Correspondence: [organicboron@ujs.edu.cn](mailto:organicboron@ujs.edu.cn)； Tel.: +82-511-8503-8201

**Figure S1** **…………………………………………………………………………………………**^1^H NMR of **2**

**Figure S2 …………………………………………………………………………………………**^13^C NMR of **2**

**Figure S3** **…………………………………………………………………………………………**^1^H NMR of **BDP-1**

**Figure S4 …………………………………………………………………………………………**^13^C NMR of **BDP-1**

**Figure S5** **…………………………………………………………………………………………**^1^H NMR of **BDP-2**

**Figure S6** **…………………………………………………………………………………………**^13^C NMR of **BDP-2**

**Figure S7** **………………………………………………………………………………………**IT-MS of compound **2**

**Figure S8** **…………………………………………………………………………………………**HR-MS of **BDP-1**

**Figure S9** **…………………………………………………………………………………………**HR-MS of **BDP-2**

**Figure S10** **…………………………………………………………………………**TEM characterizations of the products


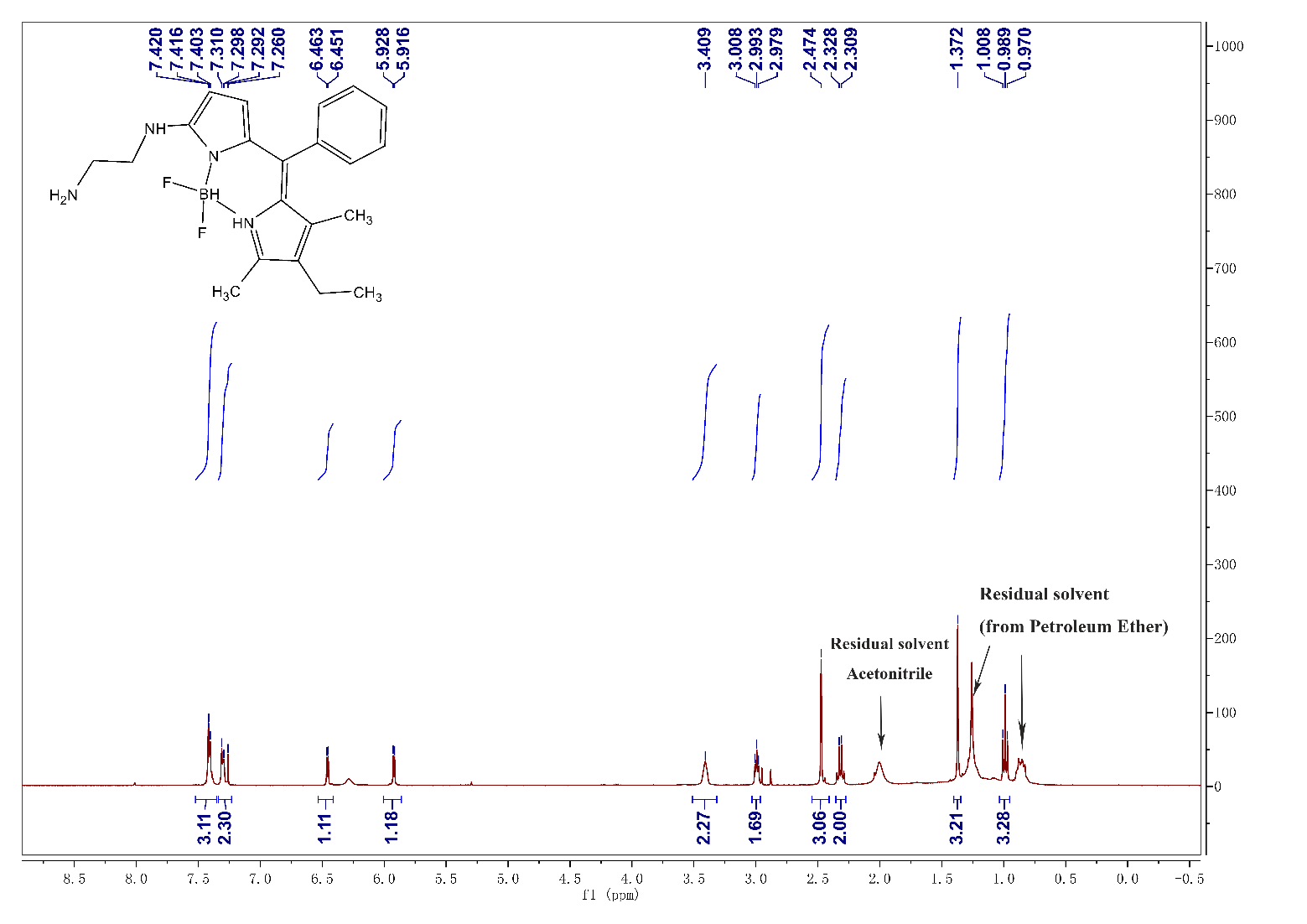


Figure S1 ^1^H NMR of compound **2**.


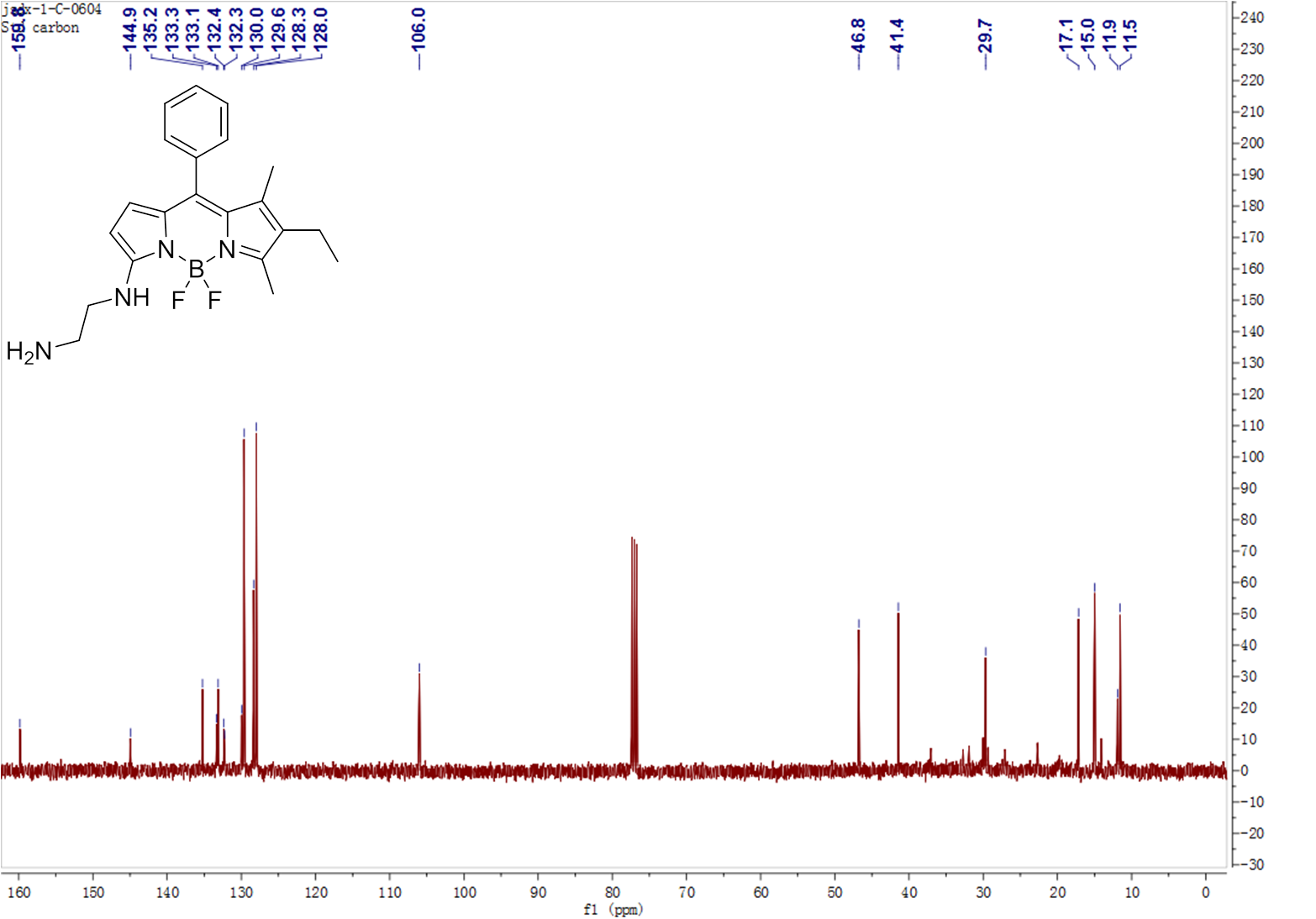


Figure S2 ^13^C NMR of compound **2**.


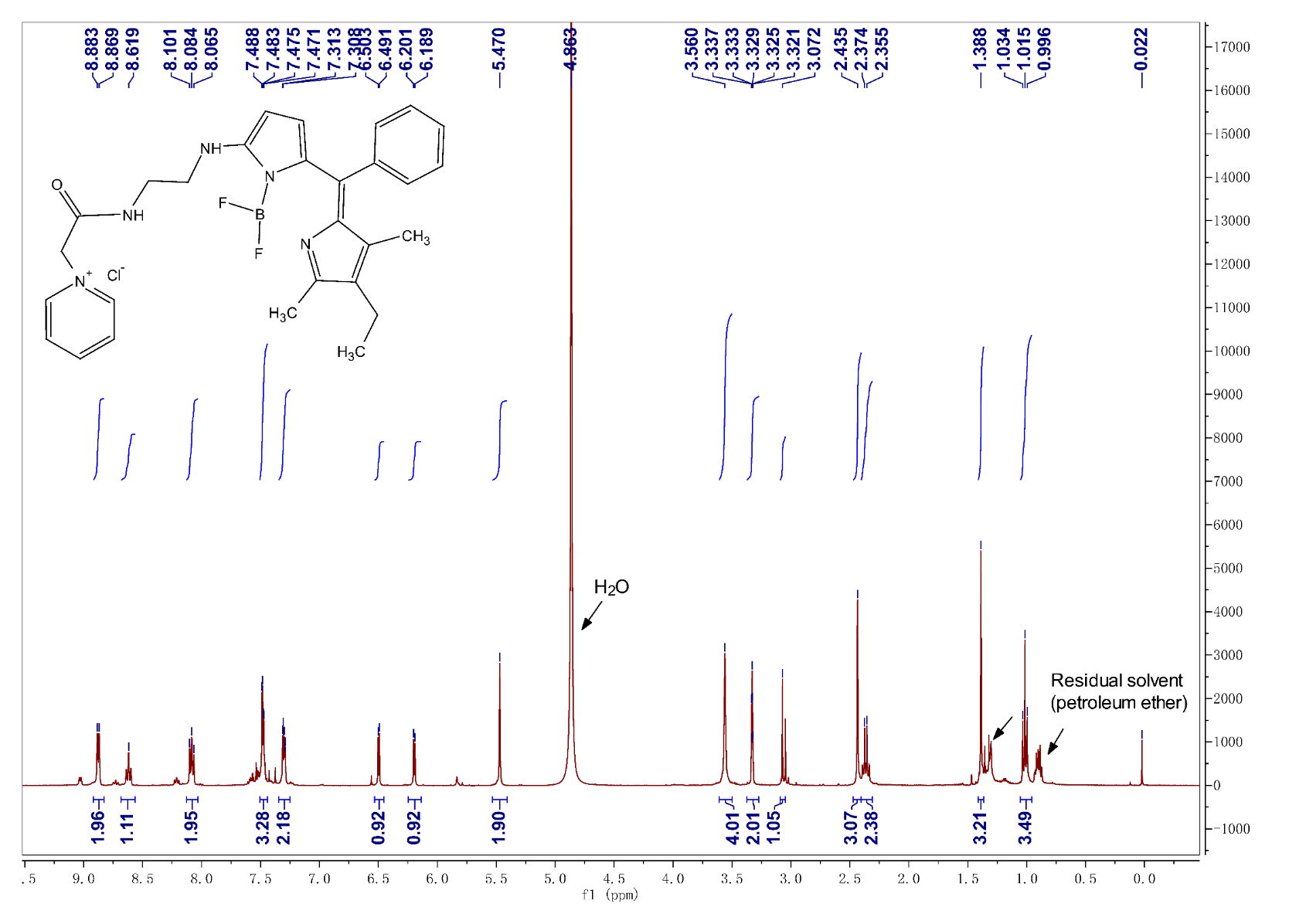


Figure S3**.** ^1^H NMR of **BDP-1**.


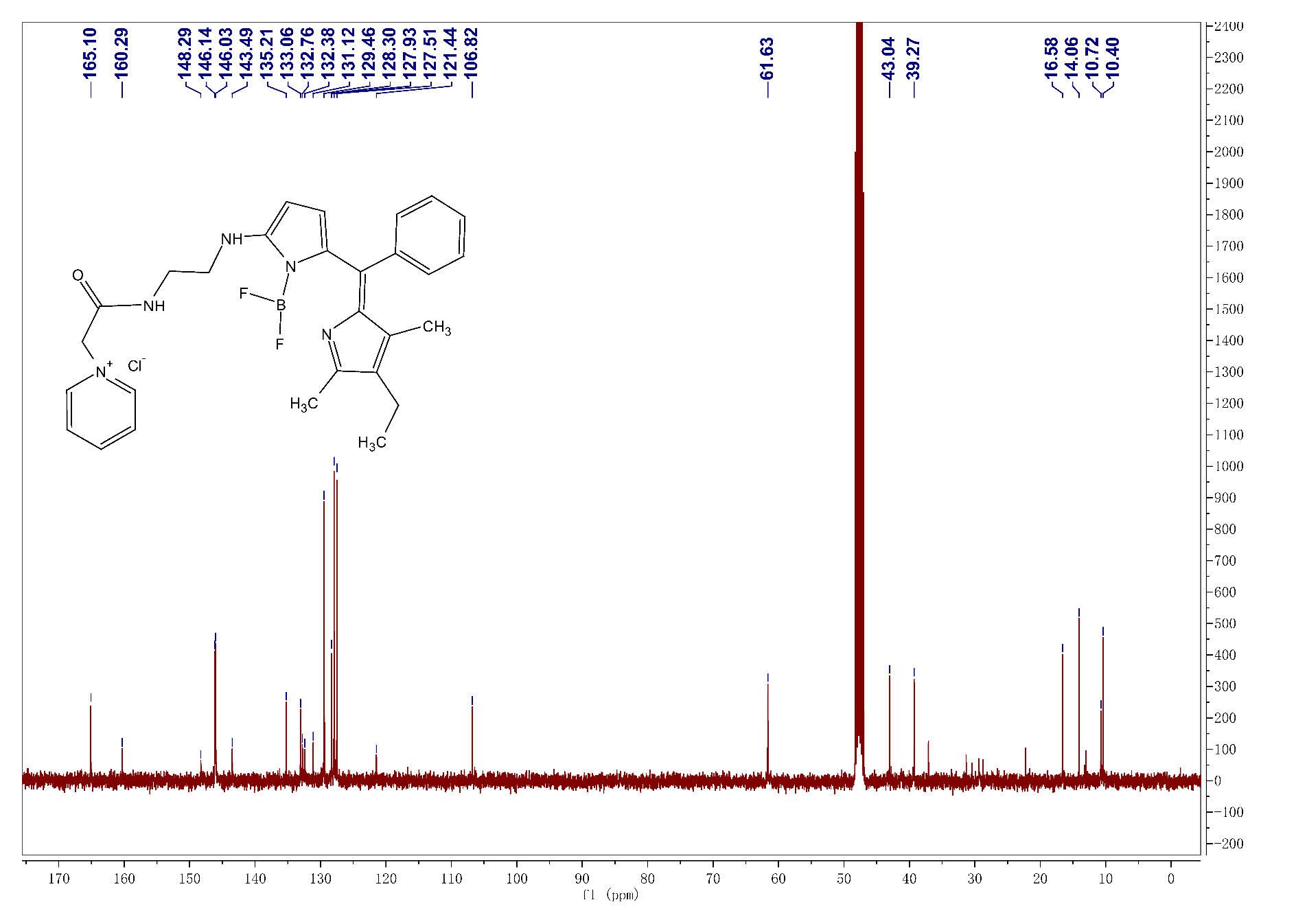


Figure S4. ^13^C NMR of **BDP-1**.


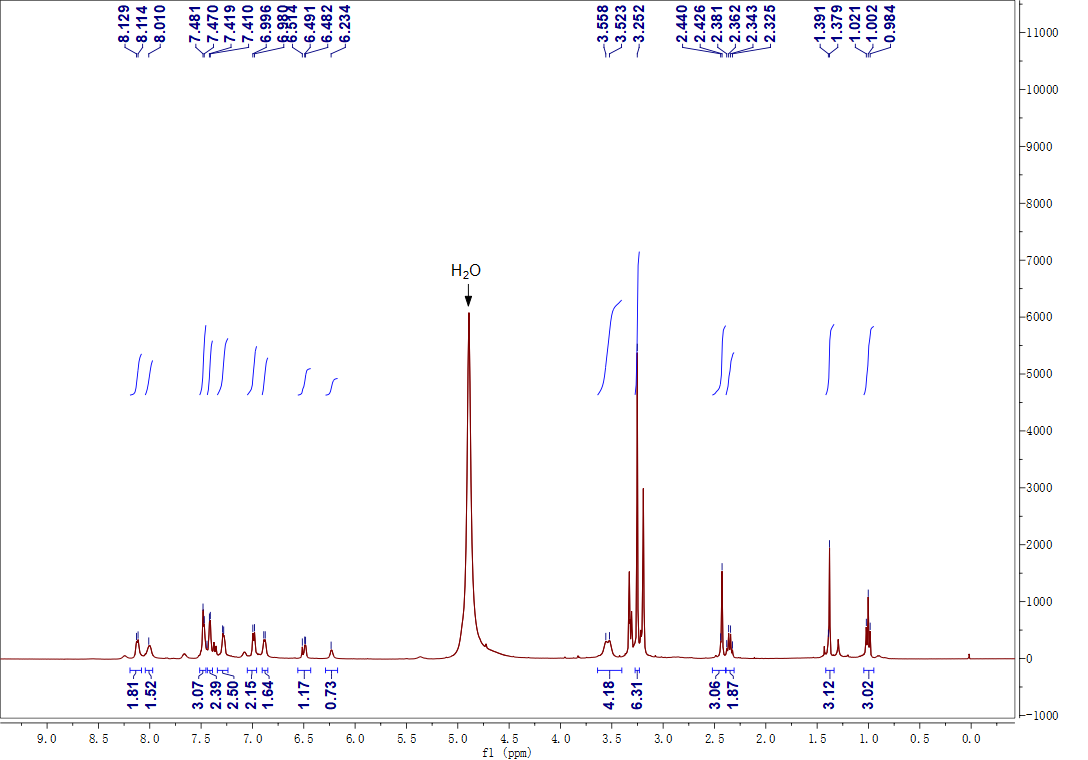


Figure S5**.** ^1^H NMR of **BDP-2**.


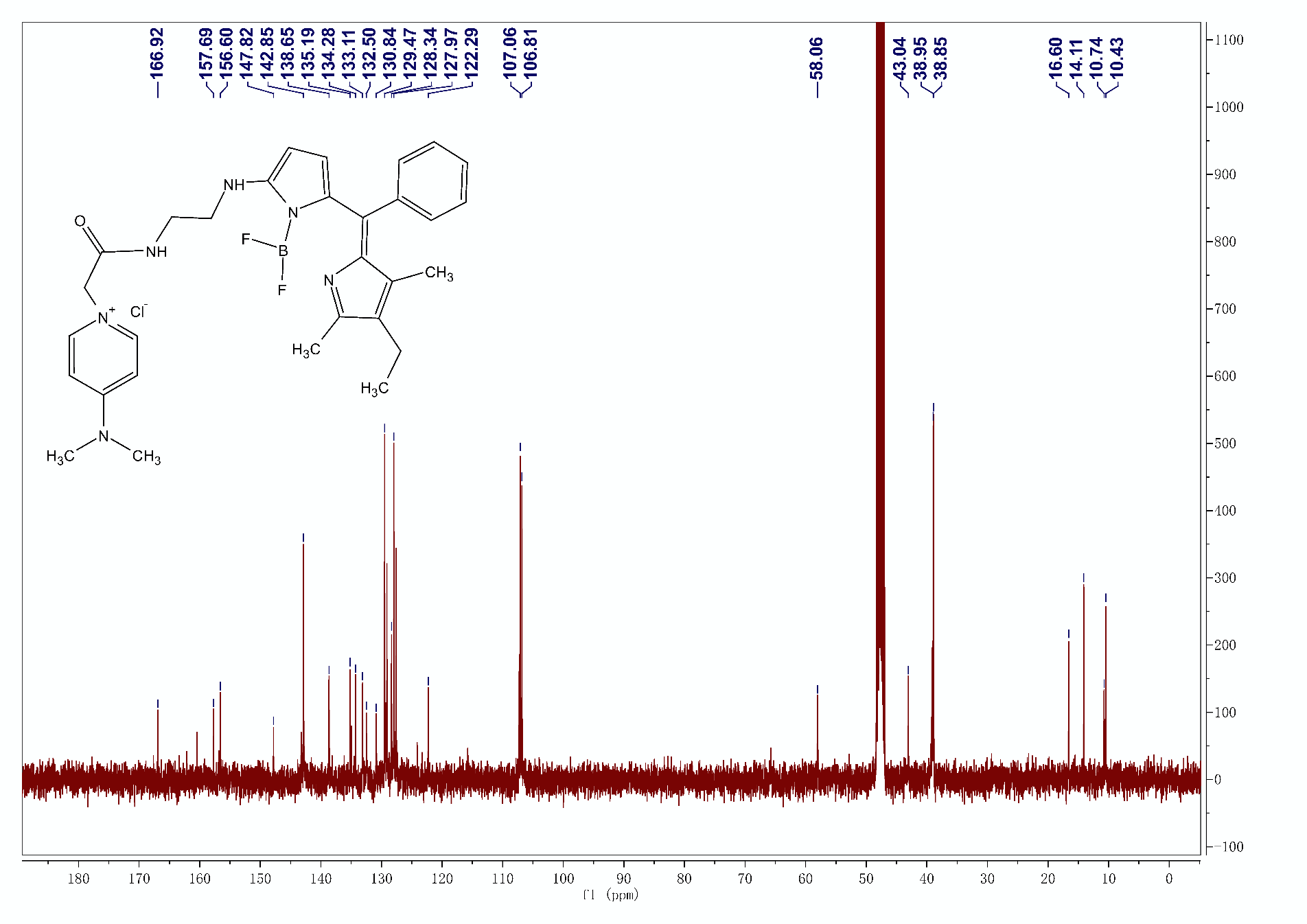


Figure S6. ^13^C NMR of **BDP-2.**


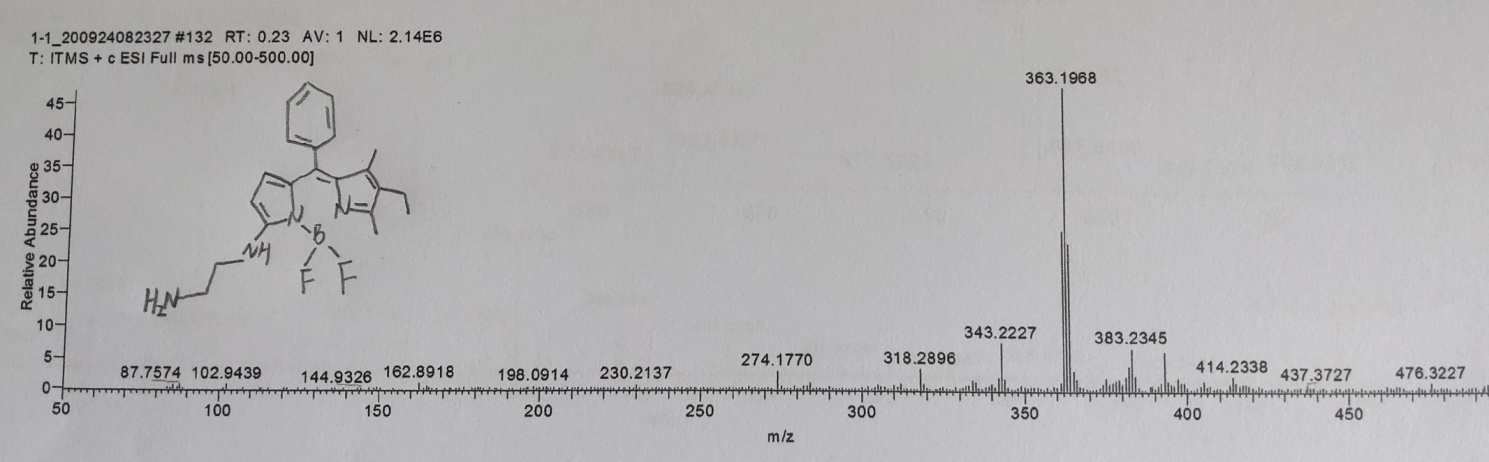


Figure S7. ITMS (ESI) of compound **2**.**
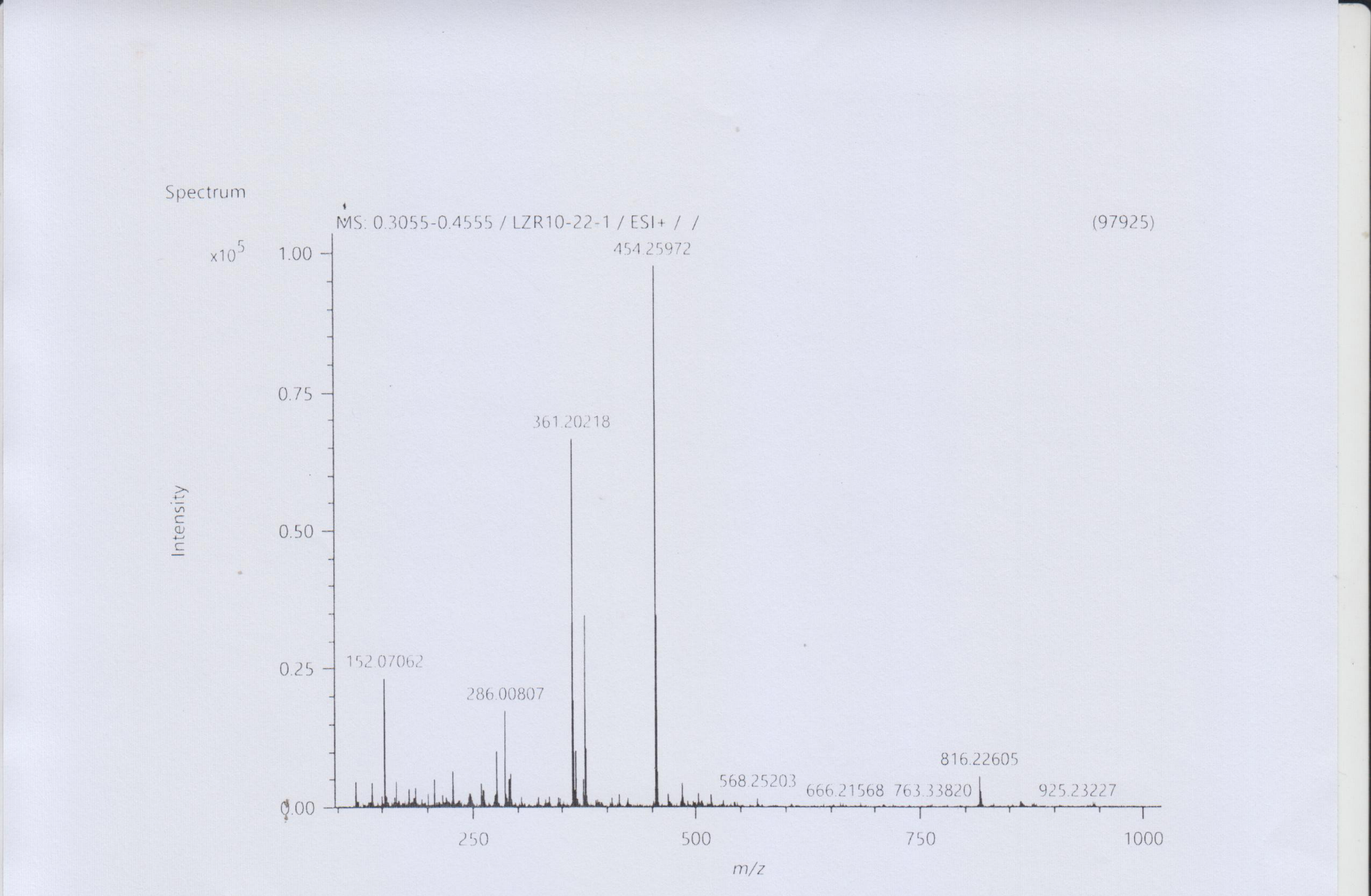
**

Figure S8**.** HR-MS of **BDP-1**.

**
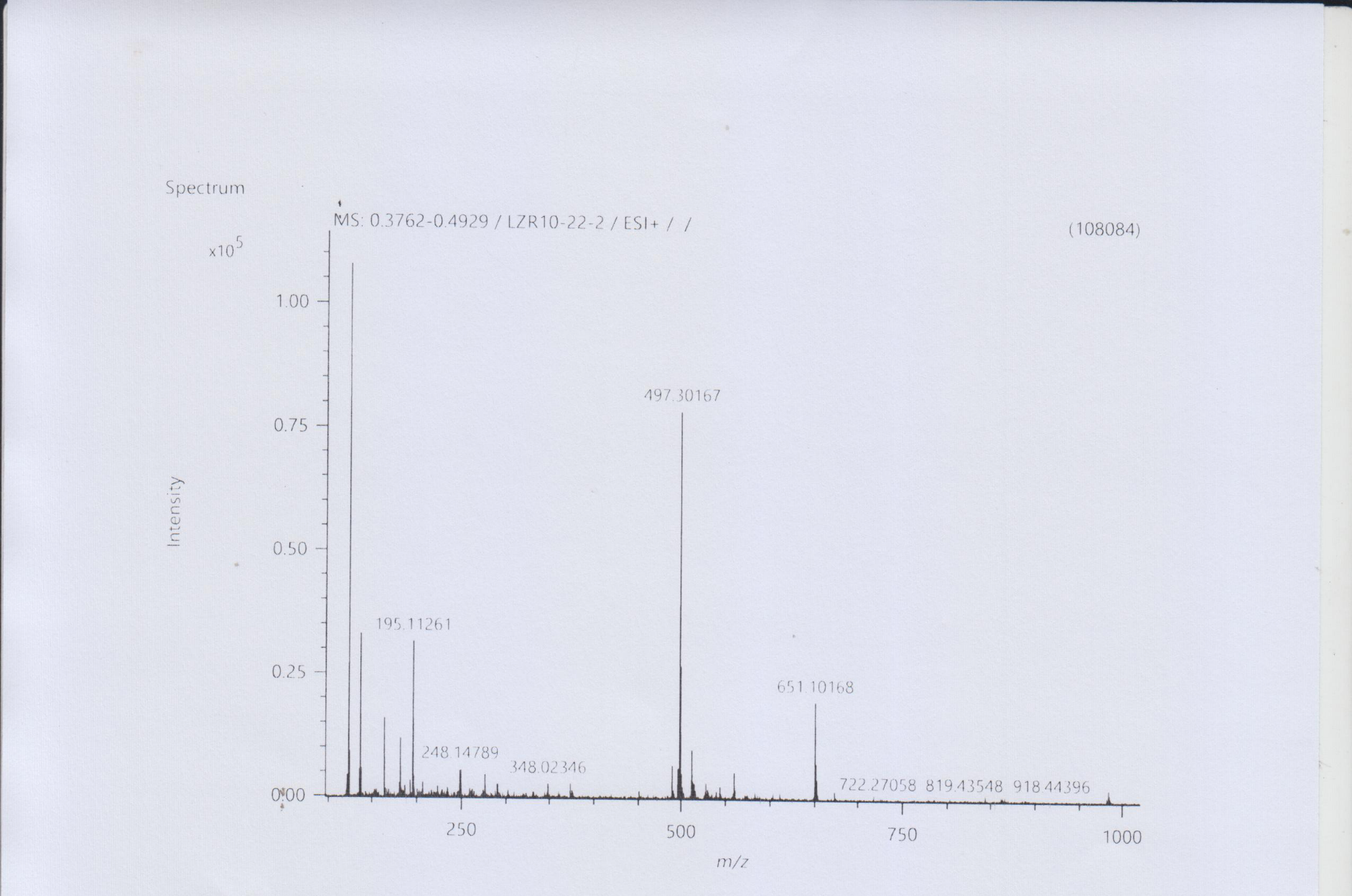
**

Figure S9. HR-MS of **BDP-2**.


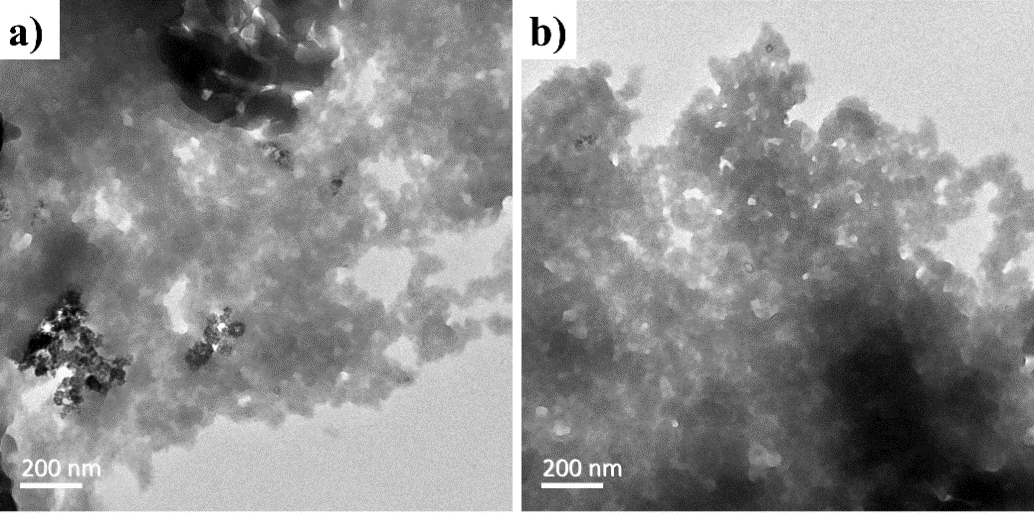


Figure S10. TEM characterizations of the products (a) BDP-1, scale bar is 200 nm; (b) BDP-2, scale bar is 200 nm by TEM.
